# Investigation of intervertebral disc degeneration using multivariate FTIR spectroscopic imaging†
†Electronic supplementary information (ESI) available. See DOI: 10.1039/c5fd00160a



**DOI:** 10.1039/c5fd00160a

**Published:** 2016-04-08

**Authors:** Kerstin T. Mader, Mirte Peeters, Suzanne E. L. Detiger, Marco N. Helder, Theo H. Smit, Christine L. Le Maitre, Chris Sammon

**Affiliations:** a Sheffield Hallam University , Materials and Engineering Research Institute , Sheffield , S1 1WB , UK . Email: K.Mader@shu.ac.uk; b Department of Orthopaedic Surgery , VU University Medical Center , Amsterdam , The Netherlands; c Skeletal Tissue Engineering Group Amsterdam (STEGA) and MOVE Research Institute , Amsterdam , The Netherlands; d Sheffield Hallam University , Biomolecular Science Research Centre , Sheffield , S1 1WB , UK

## Abstract

Traditionally tissue samples are analysed using protein or enzyme specific stains on serial sections to build up a picture of the distribution of components contained within them. In this study we investigated the potential of multivariate curve resolution-alternating least squares (MCR-ALS) to deconvolute 2nd derivative spectra of Fourier transform infrared (FTIR) microscopic images measured in transflectance mode of goat and human paraffin embedded intervertebral disc (IVD) tissue sections, to see if this methodology can provide analogous information to that provided by immunohistochemical stains and bioassays but from a single section. MCR-ALS analysis of non-degenerate and enzymatically *in vivo* degenerated goat IVDs reveals five matrix components displaying distribution maps matching histological stains for collagen, elastin and proteoglycan (PG), as well as immunohistochemical stains for collagen type I and II. Interestingly, two components exhibiting characteristic spectral and distribution profiles of proteoglycans were found, and relative component/tissue maps of these components (labelled PG1 and PG2) showed distinct distributions in non-degenerate *versus* mildly degenerate goat samples. MCR-ALS analysis of human IVD sections resulted in comparable spectral profiles to those observed in the goat samples, highlighting the inter species transferability of the presented methodology. Multivariate FTIR image analysis of a set of 43 goat IVD sections allowed the extraction of semi-quantitative information from component/tissue gradients taken across the IVD width of collagen type I, collagen type II, PG1 and PG2. Regional component/tissue parameters were calculated and significant correlations were found between histological grades of degeneration and PG parameters (PG1: *p* = 0.0003, PG2: *p* < 0.0001); glycosaminoglycan (GAG) content and PGs (PG1: *p* = 0.0055, PG2: *p* = 0.0001); and MRI T2* measurements and PGs (PG1: *p* = 0.0021, PG2: *p* < 0.0001). Additionally, component/tissue parameters for collagen type I and II showed significant correlations with total collagen content (*p* = 0.0204, *p* = 0.0127). In conclusion, the presented findings illustrate, that the described multivariate FTIR imaging approach affords the necessary chemical specificity to be considered an important tool in the study of IVD degeneration in goat and human IVDs.

## Introduction

Low back pain (LBP) affects millions of people worldwide, and has been linked to degenerative changes of the intervertebral disc (IVD).^1,2^ The IVD is a structurally and chemically complex cartilaginous tissue consisting of distinct regions; a softer gelatinous inner core known as the nucleus pulposus (NP) and a highly organised fibrous outer region known as the annulus fibrosus (AF).^3^ The proteoglycan and collagen type II concentrations gradually decrease from the NP out towards the AF, whilst the collagen type I concentration increases.^4,5^ Additionally, the presence and importance of elastic fibres was studied and an intricate network of elastin fibres, microfibrils and collagen fibres has been found.^6^ During aging and degeneration, IVD cells produce abnormal amounts of matrix components and matrix-degrading enzymes.^7–11^ Eventually the IVD matrix composition changes, for example, the degradation and loss of proteoglycans, a change in the distribution and composition of collagens, the denaturation of collagen type II and increased decorin and fibronectin concentrations have been reported.^12,13^ On a macroscopic level these changes in matrix composition lead to changes in the structure of the IVD, the boundary between AF and NP becomes less distinct, annular lamella become irregular, bifurcating and interdigitating, and cleft and fissure formation occurs, which leads to a loss in the functionality of the IVD.^12^ In recent years a wide range of potential therapeutic strategies have been developed allowing more sophisticated designs and enhancing the success of regenerative therapies.^14–16^ It has therefore become of great interest to develop comprehensive analytical methodologies to objectively define critical characteristics of target matrices as well as to assess repair and regeneration efficacy.

Fourier transform infrared (FTIR) spectroscopy provides information about the chemical species in a sample based on the frequency of the vibrations of its covalent bonds.^17^ Additionally, using a FTIR imaging set-up it is possible to measure spatially resolved quantitative biochemical information. This together with technological developments in this field,^18^ which now allow a typical experiment to be completed and analysed within hours, make this technique a viable tool for rapid disease screening and diagnosis in pathologies where tissue biopsies are collected as part of routine diagnosis.^18^ FTIR imaging has been used for the analysis of biological samples such as cells, bone and cartilaginous tissue in its native, repaired and regenerated state.^19–25^ Different components in the extracellular matrix of connective tissue *e.g.* collagens and proteoglycans, show discriminatory spectroscopic characteristics and information about multiple species can be derived from the measurement of a single section without the need for lengthy immunohistochemical staining from multiple successive sections.^24,25^

**Fig. 1:**
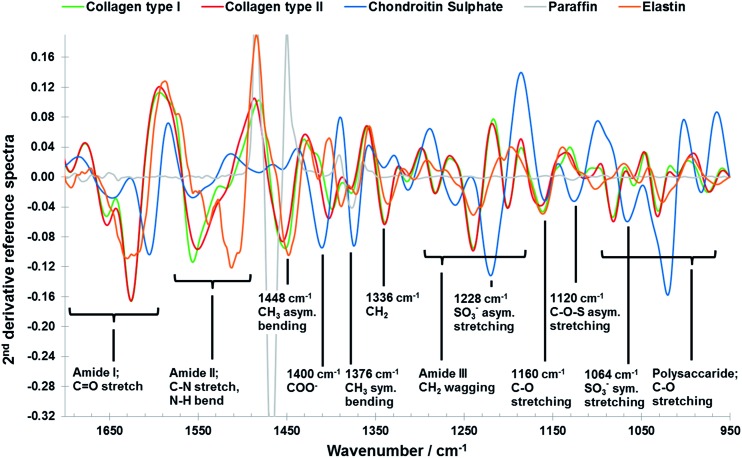
Vector normalised 2nd derivative ATR-FTIR reference spectra of collage type I (green), collagen type II (red), chondroitin sulphate (blue), paraffin (grey) and elastin (orange) components (see data collection in the materials and methods section: reference materials), including peak assignments.^43^

However, despite its great potential FTIR imaging of biological samples is not without drawbacks; significant overlap of infrared spectral profiles of different tissue components (Fig. 1) complicates the extraction of chemically specific parameters.^26^ The analysis of FTIR spectroscopic imaging data can be further hindered by spectral artefacts related to sample preparation^27,28^ and collection mode.^29,30^ This is particularly true of spectra collected in transflectance mode, which has some advantages over transmission mode, for example the use of low cost substrates, ease of sample preparation and a great potential for automation, which has been reported to be affected by spectral distortions due to reflective and optical phenomena such as Mie scattering and electric field standing wave effects.^31–38^ For FTIR spectroscopy to become a diagnostically useful tool the challenge lies in the development of analytical strategies which enable the development of robust methods with high chemical specificity. Current methodologies utilise 2nd derivative infrared spectra and multivariate analysis methods such as principal component analysis (PCA), partial least squares (PLS) and cluster analysis to enhance the data interpretation of the FTIR imaging data of cartilaginous tissue.^39–43^ Only recently the potential of multivariate curve resolution-alternating least squares (MCR-ALS) methods for the analysis of biological samples was highlighted.^44^ Like PCA, MCR methods are able to describe data matrices of multi-component measurements, without any prior knowledge of the nature and composition of the mixtures. The only assumption is a bilinear structure of the data matrices. In spectroscopic data, a bilinear structure arises through obedience of the Beer–Lambert law which implies a linear relationship between the absorbance and concentration of a component and the additivity of these absorbencies in a mixture.^45,46^ However, while PCA analysis of infrared images results in abstract factors, MCR analysis transforms abstract results into noise filtered, physically and/or chemically meaningful spectra and distribution maps of single components through the application of constraints related to the physicochemical or mathematical properties of the mixture components.^45–48^ Iterative MCR methods such as MCR-ALS have been shown to work especially well for the resolution of vibrational spectroscopic images of biological samples.^49–53^ Furthermore, MCR-ALS results can then be used as input information into segmentation analysis for histopathological classification.^44^

To date, there have been limited studies of IVDs using FTIR spectroscopy and multivariate data analysis.^54,55^ In this work, we examine the potential of MCR-ALS analysis to extract spatially resolved biochemical information of non-degenerated and enzymatically *in vivo* degenerated goat IVDs from 2nd derivative FTIR microscopic imaging data collected in transflectance mode. Additionally, the transferability of the described multivariate imaging approach from goat to human IVD sections is investigated. As a second objective, the derived methodology is applied to a second set of goat IVDs discs (*n* = 43) obtained from an *in vivo* goat study investigating the effect of two different growth factors (BMP2 and BMP2/7) in combination with a fibrin hyaluronic acid hydrogel on the regeneration of the intervertebral disc. Extracted multivariate spectroscopic parameters are correlated with parameters derived from histological grading, biochemical and MRI T2* measurements.

## Experimental

### Reference materials

Collagen type II (collagen from bovine tracheal cartilage), collagen type I (collagen from bovine achilles tendon), chondroitin sulphate A sodium salt (chondroitin sulphate A sodium salt from bovine trachea) and elastin (elastin from bovine neck ligament) were purchased from Sigma Aldrich (Gillingham, Dorset, UK). Attenuated total reflectance (ATR) FTIR spectra of reference material were collected at a spectral resolution of 4 cm^–1^ using a Nexus FTIR spectrometer (Thermo Scientific, UK) fitted with a diamond Golden Gate ATR accessory (Specac Limited, Orpington, Kent, UK). ATR-FTIR spectra were vector normalised (950–1800 cm^–1^, ISys 5.0.0.14) and second derivatives of the spectra were generated using the Savitzky–Golay transformation (filter order 3, filter length 15, ISys 5.0.0.14).

### Goat IVD samples; set one

Non-degenerated (control) and enzyme induced mildly degenerated goat IVDs were provided by the VU University Medical Centre, Amsterdam. The samples originate from a larger set of samples used to study the biomechanical and NP viscoelastic properties of non-degenerate and mildly degenerated goat IVDs. A detailed description of the experimental set-up is outlined in Detiger *et al.* ^56^ In short, eight skeletally mature female Dutch milk goats were obtained from a local farmer. During a surgical procedure three out of six lumbar IVDs in each goat were injected with chondroitinase ABC (CABC), an enzyme which cleaves proteoglycans and more specifically glycosaminoglycans (GAGs), which thus induces the onset of degeneration.^57–60^ The research protocol was approved by the Scientific Board and the Animal Ethics Committee of VUmc. To confirm that mild degeneration had occurred, radiographic and MRI analyses were conducted. After biomechanical and viscoelastic testing the samples were stored at –20 °C until further analysis. After thawing, IVDs were cut from the endplates with a surgical knife, formalin fixed (10% v/v, overnight at 4 °C) and paraffin embedded (sagittal directions). Consecutive 4 μm sections of each sample were mounted on glass slides (Lavender X-Tra Slides, Leica, Milton Keynes, UK) for histological and immunohistochemical staining, and on custom made reflective 316 stainless steel slides for FTIR imaging analysis.

#### Histology and immunohistochemistry

Sections were deparaffinised, rehydrated and subjected to H&E, Alcian blue (pH 2.5) and Masson Trichrome (Masson Trichrome with aniline blue, Bio-Optica, Milan, Italy) staining using standard protocols. Additionally, immunohistochemistry was used to visualise the distribution of collagen type I and collagen type II. For the immunohistochemical staining paraffin sections were deparaffinised, rehydrated and endogenous peroxidase blocked using hydrogen peroxide. After washing in distilled water, sections for collagen type II staining were treated with a hyaluronidase-protease enzyme antigen retrieval solution for 1 h at 37 °C (0.2% w/v protease and 2% w/v hyaluronidase in TBS; Sigma-Aldrich, Gillingham, Dorset, UK). Following washing, non-specific binding sites were blocked at room temperature for 45 minutes in 25% w/v rabbit serum (Abcam, Cambridge, UK) in 1% w/v BSA/TBS (Sigma, Gillingham, Dorset, UK). Sections were incubated overnight at 4 °C with type II collagen monoclonal primary antibody (1 : 100 dilution; Developmental Studies Hybridoma Bank, The University of Iowa, Iowa City, USA). Additional sections were treated with mouse IgG1 isotype controls (NCG01) (Abcam, Cambridge, UK) and used as negative controls. To determine collagen type I content within a section, a heat/citric acid antigen retrieval method (0.05 M Tris–HCL, pH = 9.5, microwave 5 min at 40% power followed by 5 min at 20% power (Sanyo Microwave, 900 W)) was used and non-specific binding sites were blocked at room temperature for 45 minutes with 25% w/v donkey serum in 1% w/v BSA/TBS (Sigma, Gillingham, Dorset, UK). Sections were incubated overnight at 4 °C with rabbit polyclonal to collagen type I (1 : 100 dilution; Abcam, Cambridge, UK) and rabbit polyclonal IgG isotype controls (Abcam, Cambridge, UK) were used for negative controls. On the following day all sections were incubated with secondary antibodies for 30 min at room temperature (collagen type II: 1 : 400 dilution of rabbit polyclonal secondary antibody to mouse IgG H&L (Biotin) and collagen type I: 1 : 200 dilution of Donkey anti-rabbit IgG H&L (Biotin) (Abcam, Cambridge, UK)). Following washes in TBS sections were incubated in ABC solution (R.T.U. Vectastain® Universal Elite® ABC kit, Vector Laboratories, Inc., CA, USA) for 30 minutes at room temperature. Following TBS washes, antibody staining was disclosed by incubation with 3,3′-diaminobenzidine tetrahydrochloride solution (Sigma, Gillingham, Dorset, UK) for 20 min at room temperature and counterstained with Mayers Haematoxylin (Raymond A Lamb, Eastbourne, East Sussex, UK). Sections were dehydrated and mounted in Pertex (Leica, Milton Keynes, UK).

#### FTIR imaging and data analysis

Mid-infrared microscopic images were collected using an Agilent 680-IR FTIR spectrometer coupled to an Agilent 620-IR FTIR imaging microscope. The microscope was fitted with a liquid nitrogen cooled 64 × 64 mercury–cadmium–telluride focal plane array detector (FPA) and an automated sampling stage. FTIR mosaic images (23 × 57 images, pixel aggregation 256, image pixel dimensions: 92 × 228) covering an area of approximately 8.05 × 19.95 mm of the tissue sections were collected in transflectance mode at a spectral resolution of 4 cm^–1^. Infrared data matrices of the control and injected samples were collated resulting in a 184 × 228 pixels data matrix. Data was pre-processed by performing a second derivative transformation on the spectra (Savitzky–Golay: filter order 3, filter length 15; ISys 5.0.0.14 software, Malvern Instruments Limited, Malvern, Worcestershire, UK). Pixels within the imaging data set where spectra indicated only the presence of paraffin or substrate were identified and masked using spectral statistics (histogram at 1554 cm^–1^, masked at 0; ISys 5.0.0.14). The data sets were analysed using a MCR-ALS algorithm described in detail by Wang *et al.* ^61^ The basic procedures of the MCR-ALS algorithm comprised the transformation of abstract factors deduced from non-linear iterative partial least squares (NIPALS) decomposition into chemical and concentration information by applying a modified iterative alternating least-square optimisation.^61^ MCR-ALS was carried out using the MCR-ALSv1.6 software with the following settings; decomposition method: NIPALS; maximum number of iterations: 500; soft constraints: MALS-2D (MCRv1.6 Copyright© 2003–2004 Unilever, UK). Two spectral regions 950–1300 and 950–1600 cm^–1^ were analysed using a range of four to six components in the MCR-ALS model. No spectral correction or exclusion of the spectral region ∼1400–1500 cm^–1^, which is dominated by absorbance peaks of the embedding medium paraffin, was carried out. Both paraffin and potential spectral distortions are considered as additional signal contributions during MCR-MLS resolution.^62^

### Human IVD samples

Two human cadaveric discs were obtained within 72 h of death from the Leeds tissue bank (ethical approval: H1306/98). Samples were obtained from a 45 year old male L4/5 disc and 74 year old female L1/2 disc.

#### Histology

A wedge of IVD tissue encompassing AF and NP was formalin fixed (10% v/v) and paraffin embedded. Consecutive 4 μm sections of each disc were mounted on glass slides (Lavender X-Tra Slides, Leica, Milton Keynes, UK) for histological analysis and on custom made reflective 316 stainless steel slides for FTIR imaging analysis. Sections were histologically graded between 0 and 12 based on the presence of cell clusters, fissures, loss of demarcation and haematoxophilia (indicating reduced proteoglycan content) with each component scored out of 3. A score of 0 to 3 indicates a histologically non-degenerate IVD and a grade of ≥4 indicates evidence of degeneration, as described previously by Le Maitre *et.al.* ^63^ Discs were graded as: male L4/5 disc: grade 2 (non-degenerate); female L1/2 disc: grade 8 (degenerate).

#### FTIR imaging and data analysis

FTIR mosaic images of human sections were collected covering an area of approximately 9.8 × 21.7 mm (pixel aggregation 256; image pixel dimensions: 112 × 248 pixels) and the same data analysis as described for the goat samples was applied.

### Goat IVD samples; set two

A second set of 43 IVDs were obtained from an *in vivo* goat study investigating the effect of two different growth factors (BMP2 and BMP2/7) in combination with a fibrin hyaluronic acid hydrogel on the regeneration of the intervertebral disc as reported in Peeters *et al.* ^64^ The research protocol was approved by both a Scientific Board as well as the Animal Ethics Committee of the VU University Medical Center. Seven skeletally mature female Dutch milk goats were used for this study. In the first surgical procedure, mild intervertebral disc degeneration was induced by injecting ±200 μl chondroitinase ABC (CABC) 0.25 Units per ml in the lumbar IVDs using a 29 G needle of 1 cm length. Another disc (T13-L1) was left as a non-degenerate control.^57–60^ During the second surgery, twelve weeks later, IVDs were injected with hydrogel in combination with either the BMP-2 or BMP-2/7 growth factor and one level was injected with hydrogel only. Detailed discussions about the safety and efficacy of the nanobiopolymeric fibrin–hyaluronic acid (FBG–HA) conjugated hydrogels, alone or in combination with BMP-2 or BMP2/7 growth factors are reported in Peeters *et al.* ^64^ In short it was found, that although mild degeneration was induced after injection with CABC, which was indicated by a significant disc height loss of injected IVDs, no significant differences in histological grades, MRI T2* mapping, GAG and total collagen content were found between the control and treated IVDs. However, it was found that MRI T2* mapping showed strong and significant correlations with biochemistry and histology in the NP.

#### MRI T2* relaxation time mapping

MRI scans were acquired from all lumbar spines within 2–3 hours after autopsy using a 1.5 T MR scanner (Magnetom Symphony, Syngo MR VA30; Siemens Healthcare). Sagittal scans were performed using a T2-weighted turbo spin echo sequence, followed by a multi-echo gradient echo sequence for T2* mapping (echo times 5.7, 10.9, 16.05, 21.2 and 26.4 ms).^57^ Each IVD was divided into 5 different regions of interest (ROI) covering the IVD from anterior to posterior where ROI 1 and 3 covered 27.5% of the total disc diameter whereas ROI 2,4 and 5 each covered 15% (Centricity RA 600, Radworks, USA). This way ROI 1, 3 and 5 represent the anterior annulus fibrosus (aAF), NP and posterior annulus fibrosus (pAF) respectively. ROI 2 and 4 represent the transition zones between AF and NP and were not used for analysis. Mean T2* relaxation times were calculated for each ROI fitting the signal intensities of the five echo times by a linear-log least-squares method using Microsoft Excel (Microsoft Office 2010).

#### Histology

After obtaining MRI scans, IVDs including endplate were dissected from the spine using an oscillating band saw. Mid-sagittal slices of 4 mm were cut, fixed in 4% formalin, decalcified in Kristense fluid and subsequently embedded in paraffin. Consecutive 3 and 4 μm sections of each sample were mounted on glass slides for histological staining, and on custom made reflective 316 stainless steel slides for FTIR imaging analysis respectively. Sections were stained with Alcian blue (Alcian Blue-Periodic Acid Schiff). Stained sections were scored for disc degeneration by two independent researchers using a histological grading scale adapted for goats as described by Hoogendoorn *et al.* ^59^ Differences in scoring were resolved by consensus, resulting in a final histological scoring ranging between 0 (non-degenerate) and 6 (degenerate).

#### FTIR imaging and data analysis

**Fig. 2:**
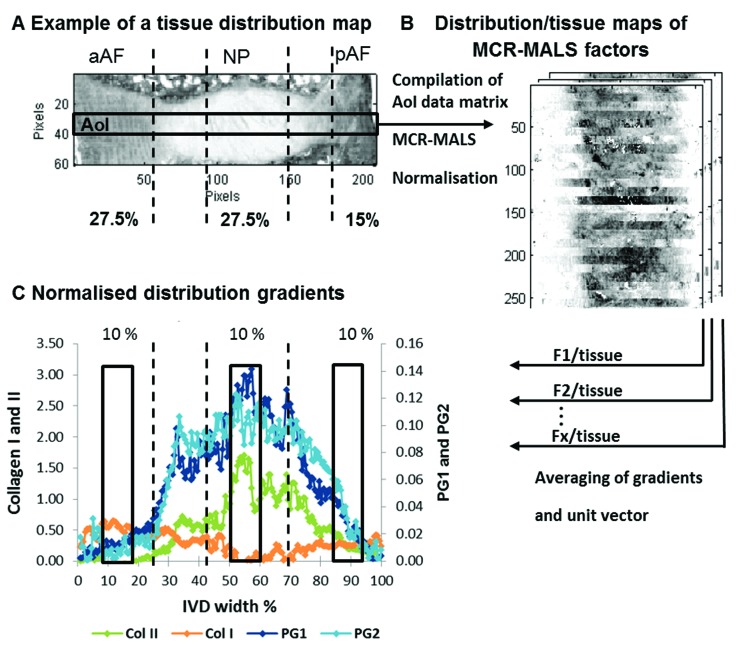
Schematic representation of the extraction of multivariate FTIR parameters. Showing an example of a tissue distribution map (peak area of the 2nd derivative of the amide III peak; 1186–1297 cm^–1^) of a paraffin embedded goat IVD section. Black lines indicate IVD regions where an IVD is divided into zones across the width of a sagittal section (described in the section MRI T2* relaxation time mapping) (A). An area of interest (AoI) across the middle of each disc spanning the whole width of each image (10 × 208 pixels) is selected and a new AoI data matrix is generated. MCR-ALS is carried out and distribution maps of selected factors are divided by tissue distribution maps to generate relative component distribution maps (B). To be able to compare IR parameters with MRI and biochemical parameters, the relative distribution maps are further processed. Firstly, the average estimated intensity across each section is taken and secondly, the actual IVD width is determined for each section and set to unity (100%). Thirdly, areas under the curve equivalent to 10% IVD width located in the aAF (10–20%), NP (51–61%) and pAF (85–95%) regions are integrated (C).

FTIR mosaic images (∼5.3 × 18.2 mm; pixel aggregation 256; image pixel dimensions: 60 × 208) of the tissue sections were collected. For the correlation of spectroscopic parameters with results from biochemical, histological and magnetic resonance imaging only areas of interest (AoI: 10 × 208 pixels) across each section (Fig. 2A) were collated for all investigated samples resulting in a 420 × 208 pixel data matrix. Data pre-treatment and multivariate analysis was performed as described in the previous FTIR imaging and data analysis section. Extracted MCR-ALS spectral profiles were assigned to collagen type I, collagen type II, and the proteoglycan parameters PG1 and PG2 according to visual matches with reference spectra and the findings as described in the results and discussion section. Relative MCR-ALS scores maps were calculated as component/tissue maps. These relative component distribution maps were further processed: firstly, the average estimated intensity across each section was taken and secondly, the actual IVD width was determined for each section and set to unity (100%) (Fig. 2B). Thirdly, areas under the curve equivalent to 10% IVD width located in the anterior AF (aAF) (10–20%), NP (51–61%) and posterior AF (pAF) (85–100%; 85–95%) regions, comparable with ROI 1,3 and 5 used for MRI T2* analysis, were integrated (ISys) (Fig. 2C).

#### Bioassays for GAG and total collagen content

Tissue samples for biochemical analysis were obtained from consecutive 4 mm slices used for histological, immunohistochemical and FTIR imaging analyses. Tissue samples were harvested from the anterior AF, NP and posterior AF analogous to ROI 1, 3 and 5 respectively, as used for MRI T2* mapping. The samples were freeze dried (speed vac) and digested in 1.5 ml papain digestion solution containing 0.1 M sodium acetate, 0.01 M l-cysteine, 0.01 M EDTA and 0.33% papain (w/v) (all Merck Millipore, USA), the pH of the papain solution was titrated to 6.6 using 1 M NaOH. Samples were digested overnight at 65 °C in a continuously shaking warm water bath. GAG content was measured using a colorimetric 1,9 dimethyl-methylene blue assay according to the manufacturer's protocol (Biolcolor Ltd., Carrickfergus, UK). Hydroxyproline (HYP) content, representing total collagen content of the tissue, was quantified using a dimethylamino-benzaldehyde assay adapted from Paul *et al.* ^65^ Both GAG and HYP were normalized by tissue dry weight (μg (mg DW)^–1^).

#### Statistical analysis

Non-parametric linear regression analysis was performed between extracted MCR-ALS parameters and histological grades, biochemical parameters and MRI T2* measurements (StatsDirect 3.0126). *P* values ≤ 0.05 were considered statistically significant and scatter diagrams were plotted using the GraphPad Prism® software (Version 6.05).

## Results and discussion

**Fig. 3:**
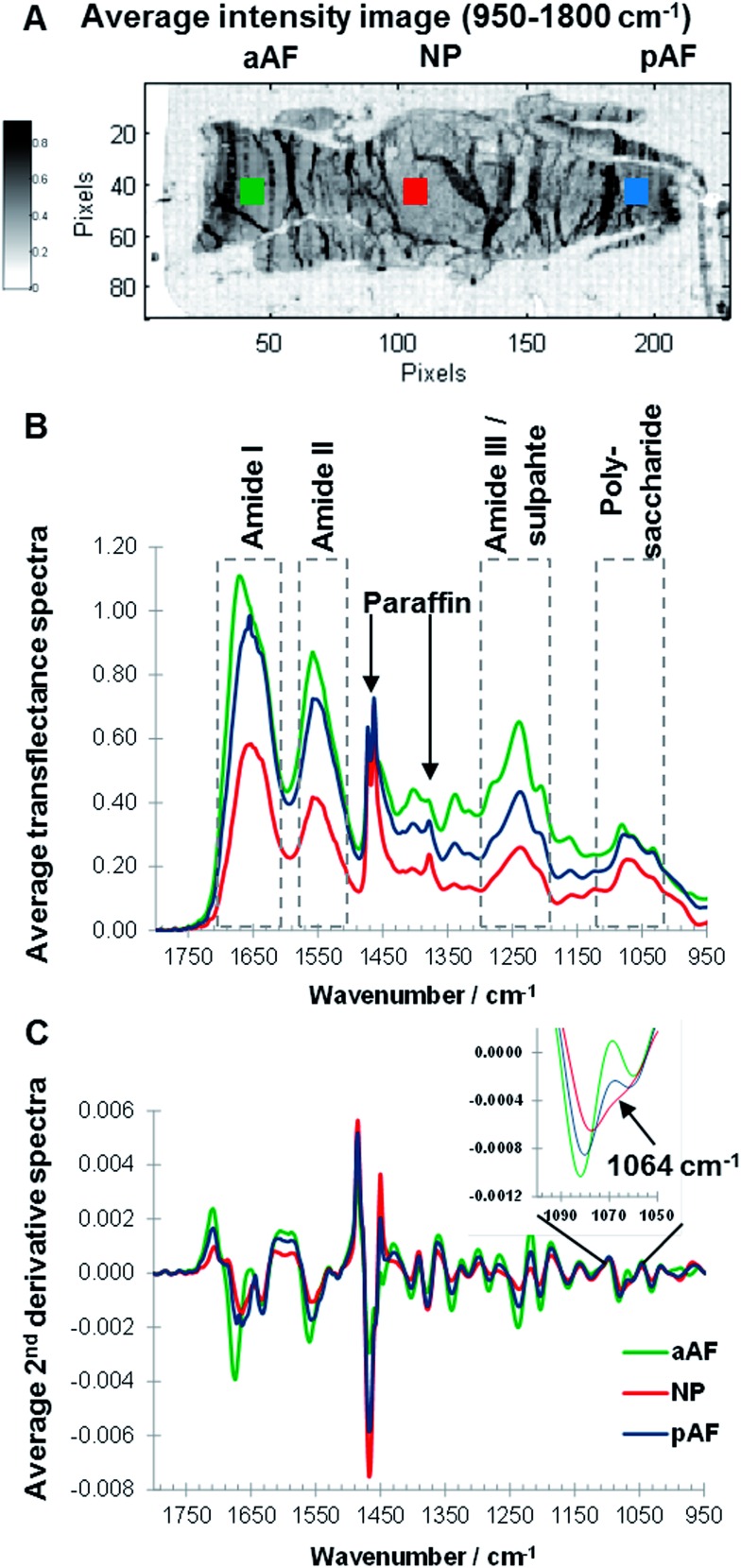
Average intensity (950–1800 cm^–1^) image of a non-degenerated paraffin embedded goat IVD section, highlighting anterior annulus fibrosus (aAF, green), posterior annulus fibrosus (pAF, blue) and nucleus pulposus (NP, red) regions (A). Off-set corrected average transflectance FTIR spectra (B) and 2nd derivative spectra (C) of aAF (green), pAF (blue) and NP(red) regions.

Sagittal sections of paraffin embedded goat IVDs were measured and an average intensity map (950–1800 cm^–1^) of a non-degenerated IVD section was generated, and the posterior AF (pAF, blue), anterior AF (aAF, green) and NP (red) regions of an IVD were highlighted (Fig. 3A). Overall the average intensity is similar across sample regions, however local higher average intensities can be observed, accentuating artificial structures such as folds formed during sample preparation. Raw spectra show considerable baseline variations (data not shown) and are off-set corrected by setting the minimum value of each spectrum (950–1800 cm^–1^) to zero (Fig. 3B). To compare spectra from different regions average spectra were calculated of the areas indicated with squares (Fig. 3A). Representative, average transflectance spectra of the aAF, pAF and NP regions show characteristic bands of collagens and PGs (amide I, amide II, amide III/sulphate and polysaccharide region) as well as characteristic bands of the embedding medium paraffin (1473, 1464 and 1378 cm^–1^). While paraffin peaks at 1473 and 1464 cm^–1^ have similar intensities across the sample regions; the amide I, amide II and amide III peaks show varying intensities. The band intensities of amide I, II and III in the aAF spectrum are approximately double the band intensities exhibited in the NP spectrum. The amide I peak of the aAF spectrum shows a peak shift to a higher wavenumber and asymmetry. The convoluted peak associated with polysaccharide species (900–1100 cm^–1^) shows a comparatively higher intensity in relation to the amide bands in the pAF and NP spectra, indicating a higher concentration of PGs in the pAF and NP regions. Performing a second derivative of FTIR spectra can enhance the discrimination of broad overlapping bands,^29,36^ and more detailed differences between the aAF, pAF and NP regions, especially in the polysaccharide region are revealed. Average second derivative spectra are shown in Fig. 3C and an expansion of the polysaccharide region shows a clear discrimination between the spectra, based on the negative intensity at ∼1064 cm^–1^. The second derivative amide II bands in the pAF and aAF spectra show a subtle shift to higher wavenumbers compared to the NP spectrum. The amide I band of the aAF spectrum shows a different peak shape and a shift to higher wavenumbers. Paraffin embedded goat IVD sections measured in transflectance mode show region dependent changes in spectral intensity leading to a saturation of the signal in some sample areas. Based on these findings the amide I spectral region traditionally used as a biomarker for collagens^25^ was excluded from further analysis and MCR-ALS deconvolution was carried out in the spectral region 950–1600 cm^–1^.

### MCR-ALS analysis

The number of components, or factors, used for the MCR-ALS resolution should not be a critical parameter, studying the evolution of extracted spectral profiles and corresponding distribution maps over a range of factors is good practice and can give additional information about the data set and help with the data interpretation.^47^ Details of all MCR-ALS results derived from the spectral regions 950–1600 cm^–1^ and 950–1300 cm^–1^ modelled using 4–6 factors can be found in ESI (Fig. S1 and S2†). While a detailed discussion of the MCR-ALS results is beyond the scope of this manuscript, the major findings are summarised.

**Fig. 4:**
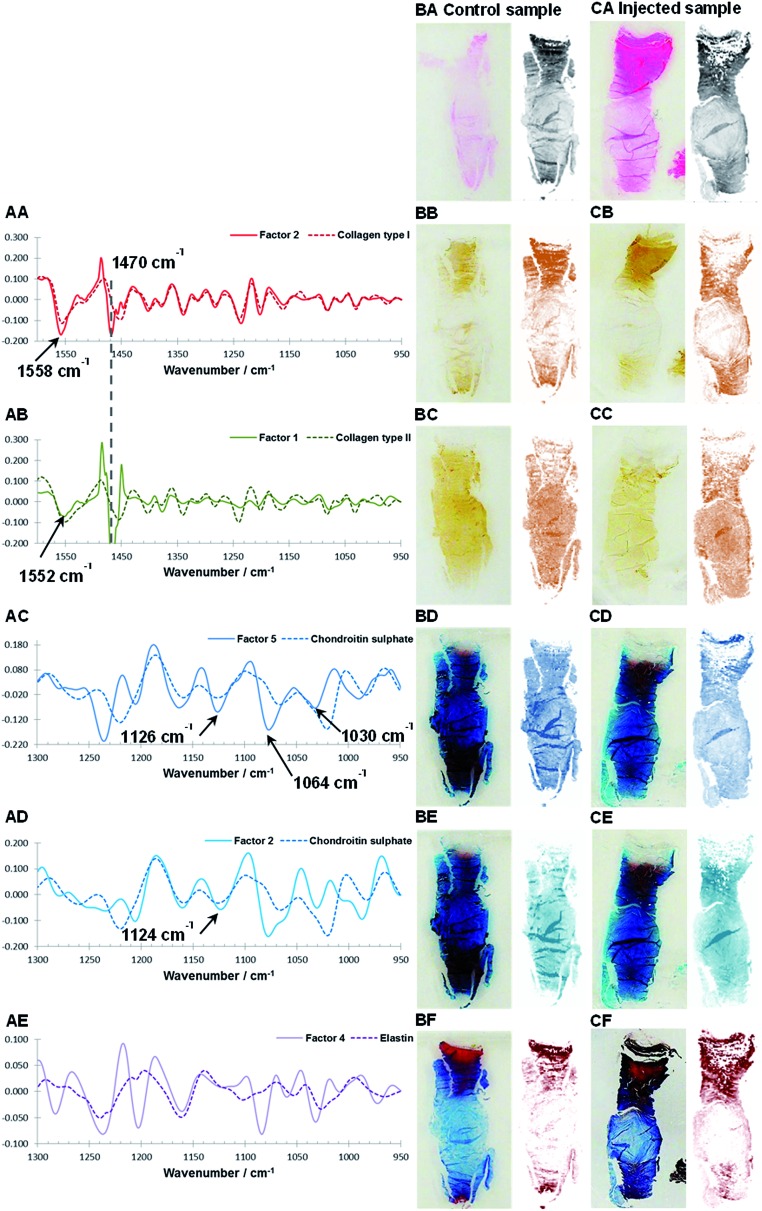
Comparison of reference spectra and extracted spectral profiles after MCR-ALS decomposition of Factor 2 (950–1600 cm^–1^) and collagen type I (AA), Factor 1 (950–1600 cm^–1^) and collagen type II (AB), Factor 5 (950–1300 cm^–1^) and chondroitin sulphate (AC), Factor 2 (950–1300 cm^–1^) and chondroitin sulphate (AD) and Factor 4 (950–1300 cm^–1^) and elastin (AE). Consecutive sagittal sections of a non-degenerated and a mildly degenerated goat IVD stained with H&E (general structure: cytoplasm and extracellular matrix: pink, cell nuclei: purple) (BA, CA), collagen type I (BB, CB), collagen type II (BC, CC), Alican blue (PGs: blue stain) (BD, CD and BE, CE) and Masson Trichrome (collagens: blue, other connective tissues *e.g.* keratin and elastin: red) (BF, CF) are shown at the left. Matching tissue distribution (BA, CA) and MCR-ALS distribution maps are shown at the right as follows: Factor 2 (950–1600 cm^–1^) (BB, CB), Factor 1 (950–1600 cm^–1^) (BC, CC), Factor 5 (950–1300 cm^–1^) (BD, CD), Factor 2 (950–1300 cm^–1^) (BE, CE) and Factor 4 (950–1300 cm^–1^) (CF, CF).

Theoretically, MCR-ALS analysis yields spectral profiles of single/pure components and in order to assist with the assignment of the biochemical identity of the extracted factors, estimated spectral profiles are compared to reference spectra of single components. However within this work the extracted spectral profiles may deviate from reference spectra for a number of reasons: (I) reference spectra were measured with ATR-FTIR, in comparison IVD sections were measured using FTIR transflectance microscopy meaning that the relative peak heights and peak positions^66^ would differ due to the non-linear effective sampling depth of the ATR measurement. (II) Intermolecular interactions between matrix components can lead to peak shifts in the spectral profiles.^17^ (III) Iterative MCR-ALS deconvolution leads to a set of feasible solution and the final results depend on the initial estimates and chosen optimisation processes.^47^ In this work, the MCR-ALS deconvolution is carried out without any input of *a priori* information such as the spectral or distribution characteristics of an IVD. Initial estimates are based on abstract factors derived from *eigenanalysis*. The four most commonly used mathematical methods to perform *eigenanalysis* are singular value decomposition (SVD), NIPALS, the power method and the Jacobi method.^46^ Although these techniques use different approaches to extract abstract factors the results should be the same.^46^ Initial investigations of loadings and scores derived from SVD and NIPALS deconvolution of the spectral regions 950–1600 cm^–1^ and 950–1300 cm^–1^ modelled using 5 factors, however showed that results can vary (S3 and S4). Nevertheless, it was found that after iterative partial least squares optimisation both sets of abstract factors lead to almost identical MCR-ALS solutions. Further inspections of the extracted MCR-ALS spectral profiles showed deviations from the reference spectra, including increased/decreased relative peak heights. One possible explanation for the observed differences could be that the used combination of abstract factors as initial estimates together with the modified iterative alternating least-square optimisation^61^ leads to spectral profiles, which emphasise the biggest spectral change in the data set rather than pure spectral profiles. For example, this could explain the observed differences between the extracted spectral profiles for PGs and the reference spectrum of chondroitin sulphate (Fig. 4AC and AD, 6BE and BF). Both natural degeneration and enzymatic degeneration induced by CABC causes a breakdown and loss of chondroitin sulphate.^12,13,59,60^ Therefore a linear decrease of all the characteristic peaks of chondroitin sulphate (*e.g.* 1376, 1228, 1120, 1064 and 1030 cm^–1^) would be expected.^43^ However the extracted spectral profiles accentuate the peak at 1064 cm^–1^ previously found to show the biggest relative changes in the investigated wavenumber region before and after enzymatic removal of chondroitin sulphate.^43^ (IV) In spectroscopic data matrices linear or near-linear relationships within a set of variables, also referred to as co-linearity, can affect the deconvolution power and stability of the MCR-ALS model. Examples of incomplete deconvolution can be seen in the spectral profiles of collagen type I and II, which show contributions of a strong peak at ∼1470 cm^–1^ characteristic of paraffin (Fig. 4AA and AB). Although, extracted spectral profiles of collagen type I and II show contributions of paraffin, corresponding distribution maps show markedly different distributions in comparison to the paraffin distribution. Furthermore, it was found that the MCR-ALS results are stable when modelled over a range of four to six factors (Fig. S1†). (V) Finally, in transflectance mode the lateral structure in heterogeneous samples leads to significant distortions of the measured spectra^34^ arising from a combination of reflective and optical factors such as a coupling between wavelengths, sample geometry, optical properties within the sample, the presence of interfaces, and the optical setup.^34^ While artefacts caused by Mie scattering can be effectively removed through the application of correction algorithms,^35,36^ spectral artefacts caused by electric field standing waves have been reported to have a detrimental effect on the sensitivity of transflectance measurements to detect biochemical differences and can limit the transferability of spectral biomarkers from one measurement mode to another.^29^

### Results of multivariate FTIR imaging of goat IVD sections (set one): comparison with histological and immunohistological stains

As discussed in the previous section, because of differences between the extracted spectral profiles and collected reference spectra, the biochemical identity of each MCR-ALS factor was established based on a visual assessment, firstly on the correlation between the reference spectra and extracted spectral profiles (Fig. 4A), and secondly by a comparison between the histological and immunohistochemical stains and the estimated distribution maps (Fig. 4B and C). The best MCR-ALS results were selected from both investigated spectral regions, and overall five MCR-ALS factors showing distinct distribution maps and spectral profiles were chosen for further discussion (Table 1).

**Table 1:** MCR-ALS resolution of the data was carried out in two spectral regions 950–1300 cm^–1^ and 950–1600 cm^–1^ using a range of four to six components. Best matches based on the visual assessment of the correlation between histological and immunohistochemical stains and estimated distribution maps as well as reference spectra and extracted spectral profiles were selected as follows

Component	Wavenumber region [cm^–1^]	No. of factors used for the model	Factor index after MCR
Collagen I	950–1600	5	2
Collagen II	950–1600	5	1
Elastic fibres	950–1300	5	4
PG 1	950–1300	5	5
PG 2	950–1300	5	2

Fig. 4BA to BF and CA to CF show consecutive sections of a non-degenerated and a mildly degenerated goat sample stained with H&E, collagen type I, collagen type II, Alcian blue and Masson Trichrome. Please note that a pixel by pixel comparison of the distribution of stained biomolecules between stains as well as stains and MCR-ALS distribution maps is not possible as the consecutive sections have slightly different shapes and sizes due to sample preparation and different staining protocols. Nevertheless, the macroscopic comparison of stains and extracted distributions is a good initial validation method. Second derivative peak integration maps of the amide III spectral region (1186–1297 cm^–1^) are proposed as a measure of overall extracellular matrix or tissue structure and match H&E stained tissue sections. Like the H&E stained sections, tissue distribution maps highlight the overall structure of an IVD (Fig. 4BA and CA). Sections show a densely packed aAF region at the top, a smaller less dense pAF region at the bottom and an only lightly stained NP region in the middle. The distribution map of Factor 2 extracted from the spectral region 950–1600 cm^–1^ shows a gradual increase of intensity from the NP towards the AF typical for collagen type I distribution in IVDs and matches well with the immunohistochemical stained section (Fig. 4BB and CB). The distribution map of Factor 1 (950–1600 cm^–1^) shows a decrease in intensity from the NP towards the AF typical for collagen type II (Fig. 4BC and CC). As discussed in the previous section, both collagen type I and type II spectral profiles show contributions of a paraffin peak at ∼1470 cm^–1^. Additionally, a peak shift of the amide II band from 1558 cm^–1^ in collagen type I to 1552 cm^–1^ in collagen type II can be observed. Distribution maps of Factor 5 (950–1300 cm^–1^) and Factor 2 (950–1300 cm^–1^) show a decrease in intensity from the NP region towards the AF region and are in good agreement with the Alcian blue stain (Fig. 4BD, CD and BE, CE). Both spectral profiles show characteristic PG peaks at ∼1030, 1064, 1160 and 1124 cm^–1^.^43^ See also the results and discussion section: MCR-ALS analysis for further comments concerning the comparison between reference spectra and extracted PG signatures. The distribution map of Factor 4 (950–1300 cm^–1^) shows a high intensity in the AF regions and matches well with the red areas of the Masson Trichrome stained sections. The calculated spectral profile of this factor resembles the reference spectrum of elastin (Fig. 4AE). Yet, while there is a good agreement between extracted distribution maps and staining, further validation of the biochemical identity of this factor against more molecular specific stains for elastic fibres is necessary.

**Fig. 5:**
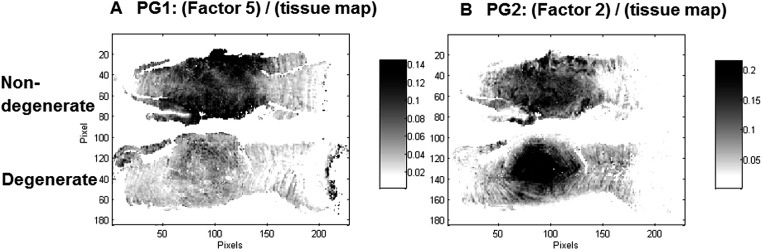
PG/(tissue map) ratios are proposed as a measure of relative PG content and distribution maps of PG1: (Factor 5)/(tissue map) (A) and PG2: (Factor 2)/(tissue map) (B) extracted from the spectral region 950–1300 cm^–1^ modelled with five factors are shown for the control (top) and injected (bottom) goat IVD samples.

Interestingly, two factors were observed (PG1: Factor 5 (950–1300 cm^–1^) and PG2: Factor 2 (950–1300 cm^–1^)), which show characteristic spectral and distribution maps of PGs. Information about PGs and more specifically aggrecan and GAG content in the NP, is of particular interest as it can be directly related to disc degeneration.^67^ PG/amide I ratios have previously been used as a measure of relative PG content in cartilage.^21^ In this work PG distributions are normalised with second derivative peak integration maps of the amide III spectral region (1186–1297 cm^–1^) (further referred to as tissue map) and relative PG distribution maps reveal distinct PG distributions in non-degenerate *versus* degenerate samples (Fig. 5A and B). While the relative component distribution map based on PG1 is less intense in the degenerate sample in comparison to the non-degenerate sample, the relative component distribution map of PG2 is more intense in the degenerate sample in comparison to the non-degenerate sample. The described MCR-ALS signatures for the two PG factors were found in all three data sets presented in this work. Initial investigations into the nature of the two PG factors have shown reproducible differences in the extracted spectral MCR-ALS signatures of PG1 *versus* PG2, including a peak shift of the C–O–S peak to a lower wavenumber from PG1 (Factor 5, 1126 cm^–1^) to PG2 (Factor 2, 1124 cm^–1^). A peak shift of the C–O–S peak has previously been observed by Rieppo *et al.*,^43^ where the authors noted a peak shift to lower wavenumbers in samples treated with the CABC enzyme, which facilitates the breakdown of GAG chains. Based on these findings PG1 would represent the “original” PG/GAG population which is decreased in degenerated samples, while PG2 could represent an altered PG/GAG population which is increased in degenerated discs. As the goat degeneration model is mimicking the onset of degeneration, much of the long term remodelling has not yet taken place and the altered PG/GAG molecules are localised in the NP.

### Results of multivariate FTIR imaging of human IVD sections

**Fig. 6:**
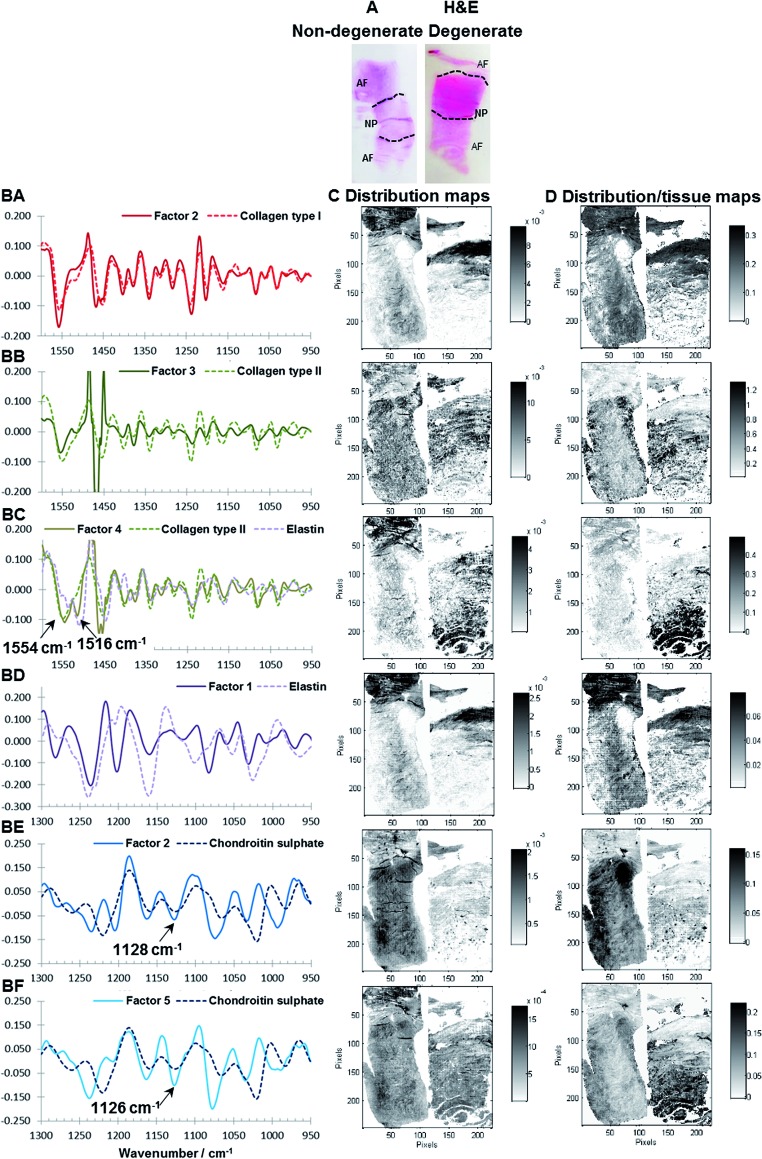
H&E of non-degenerate (left) and degenerate (right) human IVDs (A). MCR-ALS calculated spectral profiles *vs.* reference spectral profiles of Factor 2 (950–1600 cm^–1^, modelled with five factors) *vs.* collagen type I (BA), Factor 3 (950–1600 cm^–1^, modelled with five factors) *vs.* collagen type II (BB), Factor 4 (950–1600 cm^–1^, modelled with five factors) *vs.* collagen type II and elastin (BC), Factor 1 (950–1300 cm^–1^, modelled with five factors) *vs.* elastin (BD), Factor 2 (950–1300 cm^–1^, modelled with five factors) *vs.* chondroitin sulphate (BE) and Factor 5 (950–1300 cm^–1^, modelled with five factors) *vs.* chondroitin sulphate (BF) as well as corresponding distribution maps (C) and distribution/tissue maps (D) of non-degenerate (left) and degenerate (right) human IVD sections.

Extracted spectral profiles were compared with reference spectra and spectral profiles derived from the MCR-ALS analysis of goat IVDs. It was found that MCR-ALS analysis of human samples results in comparable spectral profiles as extracted from the goat samples and five factors showing similar spectral characteristics to the spectral profiles extracted for goat IVDs can be seen (Fig. 6BA–BF). H&E stains, corresponding distribution maps and distribution/tissue maps of non-degenerate (left) and degenerate (right) human discs are shown in Fig. 6A, C and D. Although the degenerate human sample is at a later stage of degeneration in comparison to the goat samples, two MCR-ALS PG signatures can be observed following the same trend; where the distribution/tissue maps of PG1 (Factor 2, 1128 cm^–1^) show a lower presence in the degenerated disc. Additionally, a localisation of PG1 within cells in the posterior AF region can be seen (Fig. 6BE). Furthermore, PG2 (Factor 5, 1126 cm^–1^) shows the highest intensity in the posterior AF region of the degenerated disc (Fig. 6BF). This region has been identified as a major location for hernias to occur and transport of PGs/GAGs into the AF region was also observed in Paul *et al.* ^65^ One additional spectral profile extracted from the spectral region 950–1600 cm^–1^ not detected in the goat sections was observed, showing two peaks at ∼1554 and 1516 cm^–1^ matching spectral characteristics of the reference spectra of both collagen and elastin (Fig. 6BC). Estimated distribution/tissue maps of this factor show a higher component content in the degenerated disc, suggesting this could be a degradation product produced during native disc degeneration in humans, and could be an additional marker for degeneration at a later stage.

### Results of multivariate FTIR imaging of goat IVD sections (set two): correlation with biochemical and MRI T2* parameters

**Fig. 7:**
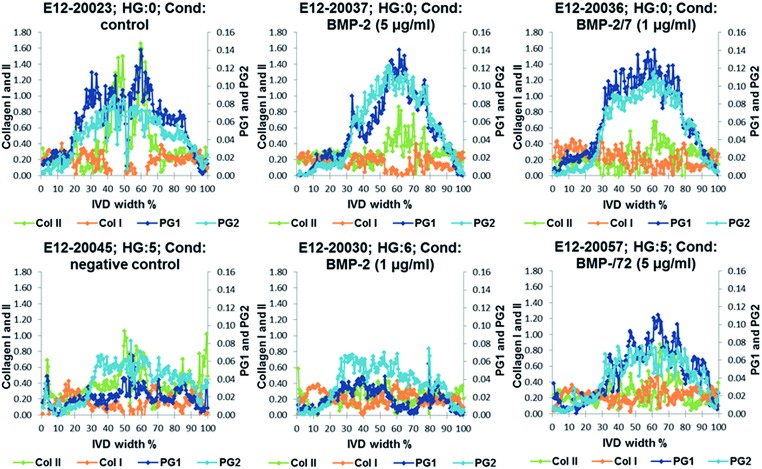
Examples of relative distribution gradients of MCR-ALS extracted factors assigned to collagen type I (Col I, orange), collagen type II (Col II, green), PG1 (dark blue) and PG2 (light blue) of samples with the lowest histological grade 0 and the two highest histological grades 5 and 6.

Relative distribution gradients for MCR-ALS extracted factors assigned to collagen type I, collagen type II, PG1 and PG2 have been compared for the lowest histological grade 0 and the two highest histological grades 5 and 6 (Fig. 7). The figure shows increasing PG and collagen type II gradients towards the NP. Both PG1 and PG2 content is markedly decreased in IVDs with a high histological grading. In the IVDs with low histological grades PG1 shows a slightly higher content in the aAF, while a slightly higher content of PG2 can be observed in discs E12-20045 and E12-20030 with histological grades 5 and 6. Additionally, a slight decrease in collagen type I content in the NP can be observed.

**Fig. 8:**
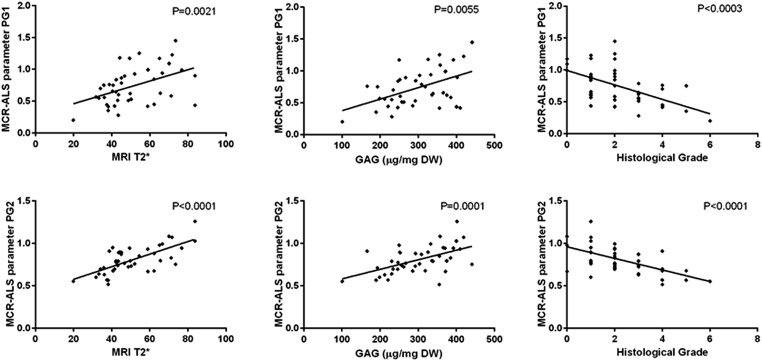
Correlation graphs between PG1 and MRI T2*, GAG content (μg (mg DW)^–1^) and histological grade (top) and PG2 and MRI T2*, GAG content (μg (mg DW)^–1^) and histological grade (bottom).

MCR-ALS extracted parameters for PG1 and PG2 were correlated with MRI T2*, GAG concentration and histological grades in the aAF, NP and pAF IVD areas. Both MCR-ALS parameters for PGs show significant correlation with the histological grades, MRI T2* and GAG content in the NP (Fig. 8) and between the MCR-ALS parameters and GAG in the pAF (PG1: *P* = 0.0017; PG2: *P* = 0.0039). Interestingly, PG2 shows a higher correlation with GAG content in the NP, while PG1 shows a higher correlation with GAG content in the pAF.

Significant correlation between collagen type I and type II and GAG content, total collagen content and MRI T2* was observed only for the NP region (Table 2).

**Table 2:** Non-parametric linear regression between histological grades, GAG content (μg (mg DW)^–1^), total collagen content (HYP (μg (mg DW)^–1^)), MRI T2* and extracted MCR-ALS matrix parameters related to proteoglycans in the IVD matrix for NP

NP	Histology	GAG (μg (mg DW)^–1^)	MRI T2*	HYP (μg (mg DW)^–1^)
Collagen type II	—	*P* = 0.0001	*P* = 0.0017	*P* = 0.0127
Collagen type I	—	*P* = 0.0022	*P* = 0.0067	*P* = 0.0204

No significant correlations were found between the MCR-ALS extracted parameters and GAG or total collagen content for the aAF region. One possible explanation might be that volumes taken for the biochemical analysis are relatively small (∼10% IVD width) and cannot be taken from exactly the same part of the IVDs used for sectioning. This is particularly relevant in the pAF, as this region is relatively large 1–27% IVD width and in comparison to the NP structurally more organised (spatially heterogeneous). This difference in sample size and position used for the different techniques may reduce the correlation between the investigated parameters. Furthermore, in this study MCR-ALS parameters for collagen type I and type II were correlated with total collagen and further studies using more specific biochemical assays are recommended.

## Conclusions

In this study, we have examined the potential of MCR-ALS analysis to extract spatially resolved biochemical information of non-degenerated and degenerated goat IVDs from 2nd derivative FTIR microscopic imaging data collected in transflectance mode. The presented results show that MCR-ALS allows the resolution of 2nd derivative FTIR microscopic imaging spectra of paraffin embedded IVD sections and important biochemical matrix components can be detected and their distribution visualised. Furthermore, matrix components specifically related to healthy and degenerate states were found. An analytical strategy was presented for the extraction of semi-quantitative biochemical information from IVD sections. Multivariate FTIR image analysis reveals the relative distribution gradients for collagen type I, collagen type II and two PG components. MCR-ALS parameters extracted from the NP region of the IVDs of both PG components show significant correlations with histology (*p* < 0.001), GAG content (*p* < 0.01) and MRI T2* measurements (*p* < 0.01). The FTIR derived parameters for collagen type I and II show significant correlations with the total collagen content (*p* = 0.02, *p* = 0.01).

Exploratory MCR-ALS results highlight the ability of multivariate curve resolution techniques to deconvolute the overall signal into single component contributions, but also the importance of understanding the molecular information contained in spectral profiles extracted using MCR-ALS analysis. Collinearity was observed in the 950–1600 cm^–1^ data set, however distinct factors for collagen type I and II can still be resolved, and good matches between the extracted spectral profiles and distribution maps with immunohistochemical stains were found.

While more molecular specific comparative experiments are recommended to further investigate the chemical nature of the extracted PG parameters and to validate correlations between the extracted collagen type I and type II parameters with collagen type I and II content, the presented results show the potential to study IVD degeneration and enable the characterisation of extracellular matrix components and their distribution in a single slice of IVD tissue, therefore allowing extensive investigations to be performed on limited tissue samples. Additionally, the described multivariate FTIR imaging method was shown to be easily transferable to human sections. The facile application across species provides a major advantage of traditional techniques such as immunohistochemistry where selective antibodies for each species are required, which involve extensive optimisation. In addition the ability of FTIR imaging to screen for variants of the extracellular matrix molecules enables identification of novel components, modifications or degradation products which is not possible *via* directed immunohistochemical analysis.
