# Supplementary material for: Investigation of intervertebral disc degeneration using multivariate FTIR spectroscopic imaging
Source: Faraday Discuss. 2016 Apr 8;187(1):393–414. doi: 10.1039/c5fd00160a (PMC5047047; doi:10.1039/c5fd00160a)
Supplement: Supplementary file 1 [file FD-187-C5FD00160A-s001.pdf]

Electronic Supplementary Material (ESI) for Faraday Discussions.
This journal is © The Royal Society of Chemistry 2016

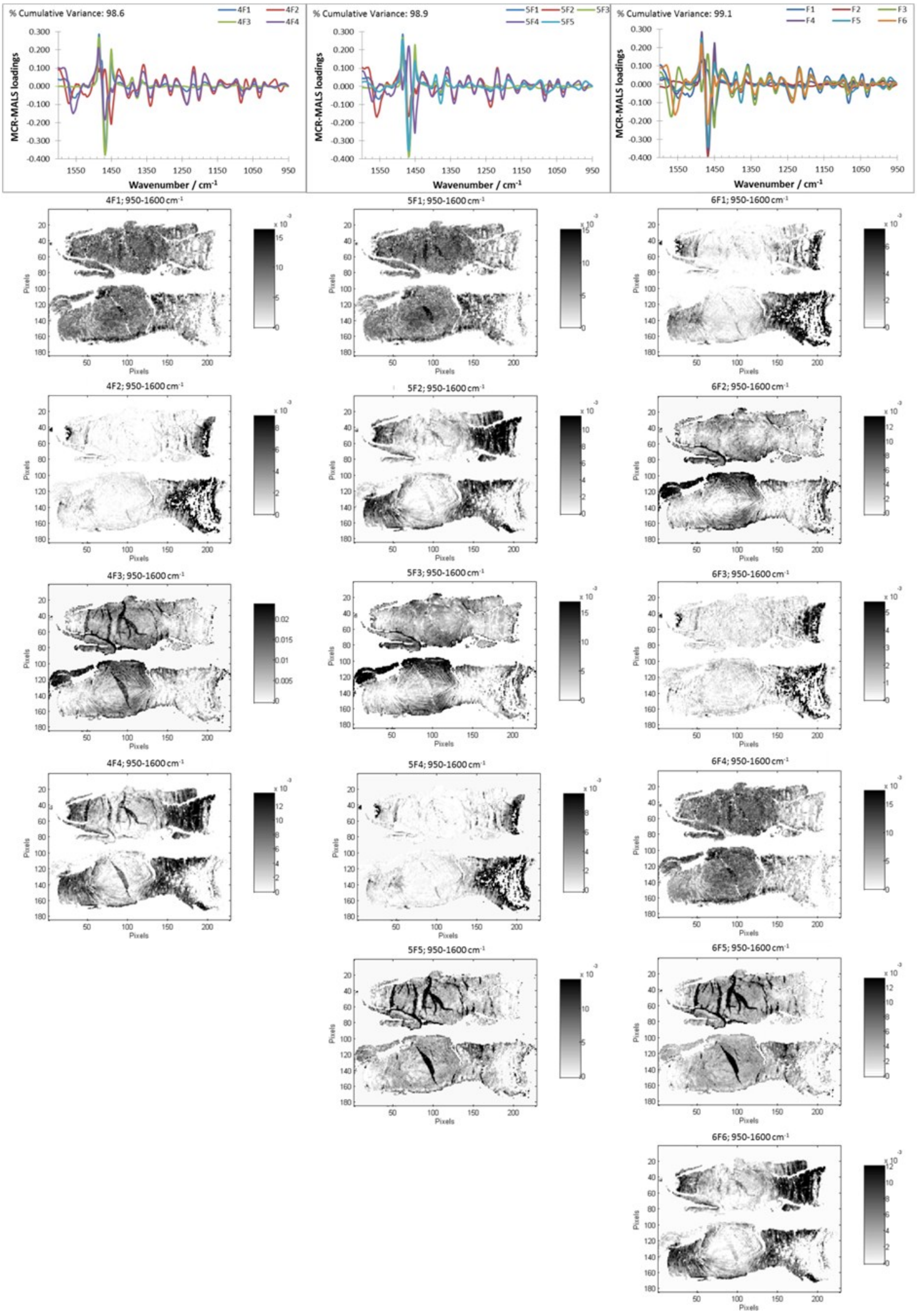

S1: Evolution of estimated spectral profiles and distribution maps derived from MCR-ALS analysis of goat IVDs samples in a wavenumber region of 950-1600 cm^{-1} using four to six factors to model the data.

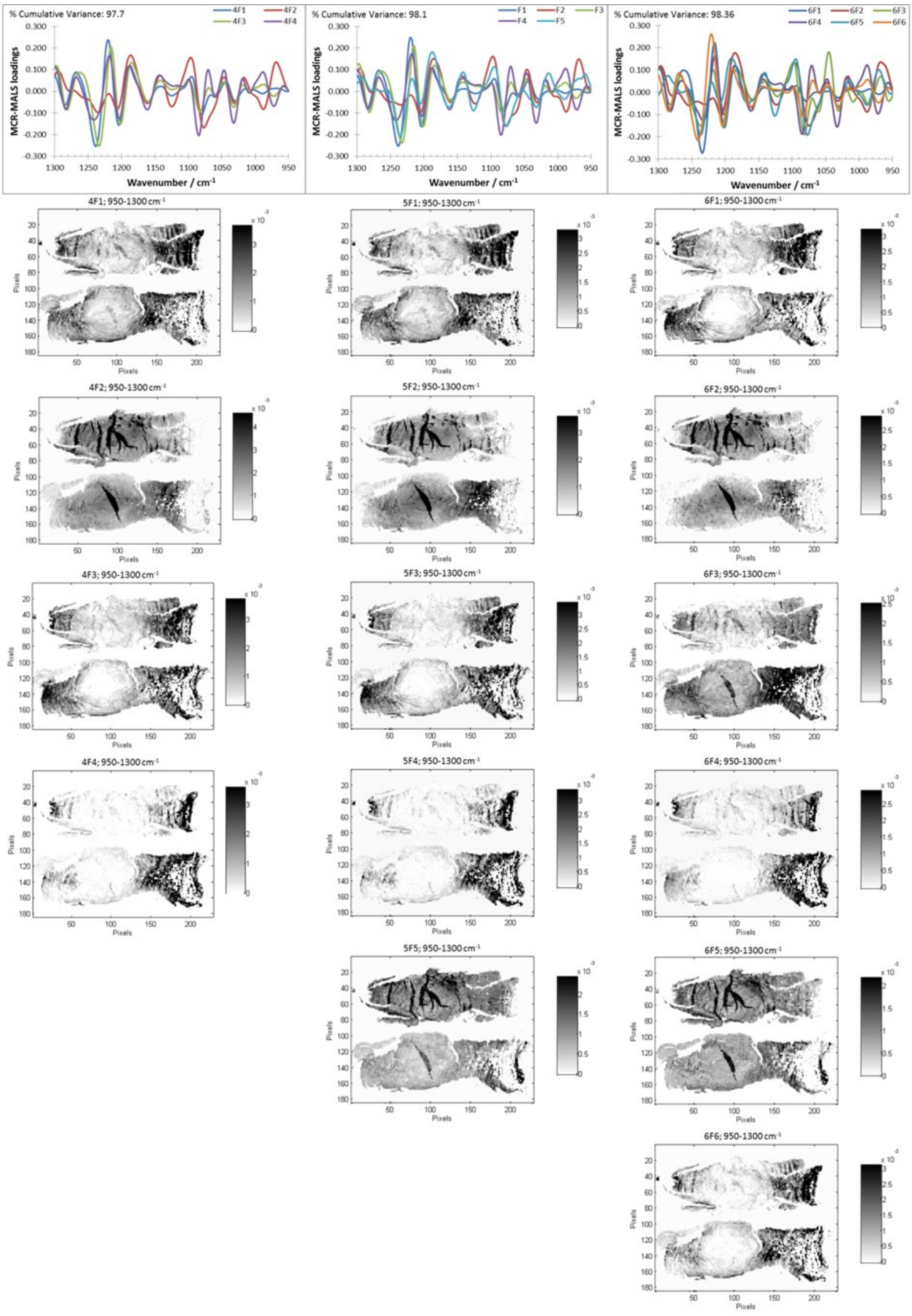

S2: Evolution of estimated spectral profiles and distribution maps derived from MCR-ALS analysis of goat IVDs samples in a wavenumber region of 950-1300 cm^{-1} using four to six factors to model the data.

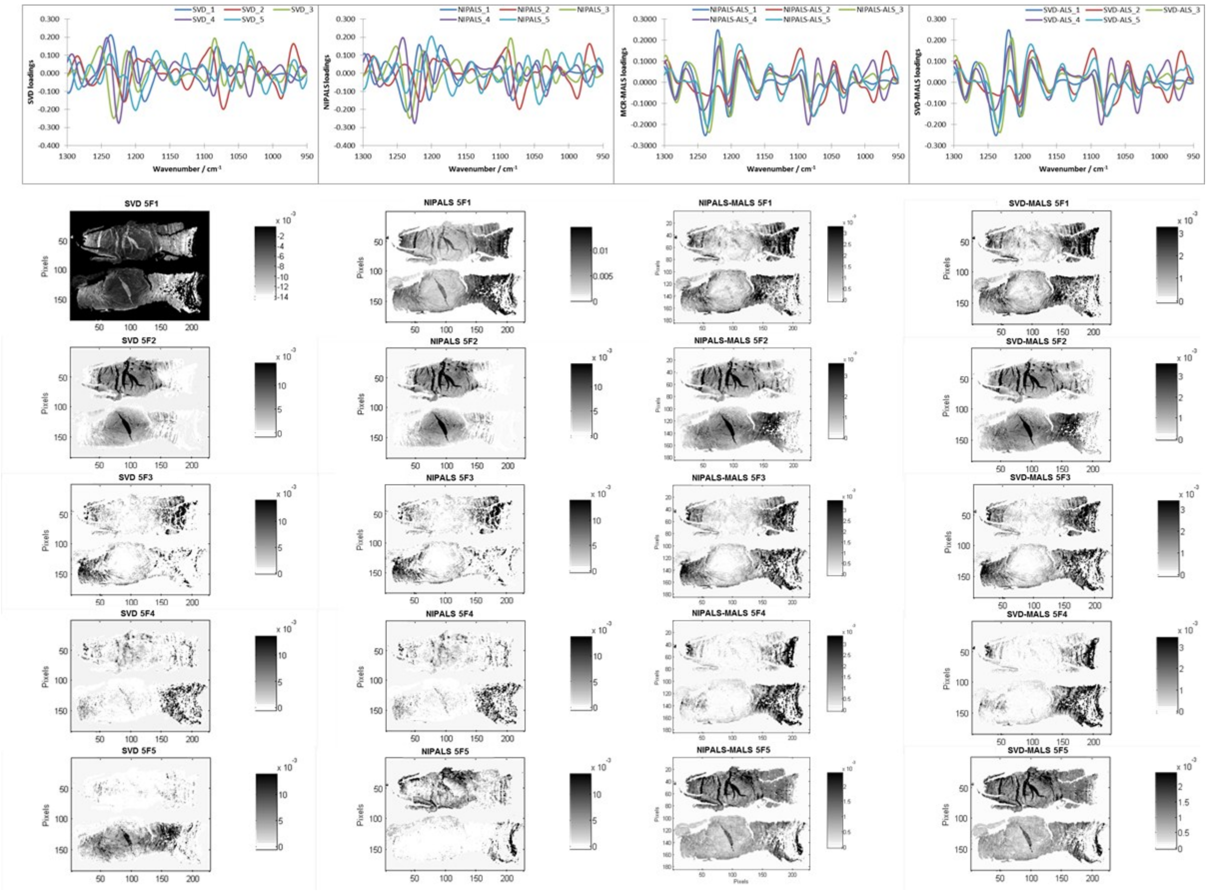

S3: Examples of loadings and scores derived from the data matrix of goat IVD samples (set one) in the spectral region of 950-1600 cm^{-1}; calculated using SVD, NIPALS as well as NIPLAS-MALS and SVD-MALS methods. All calculations are based on five components.

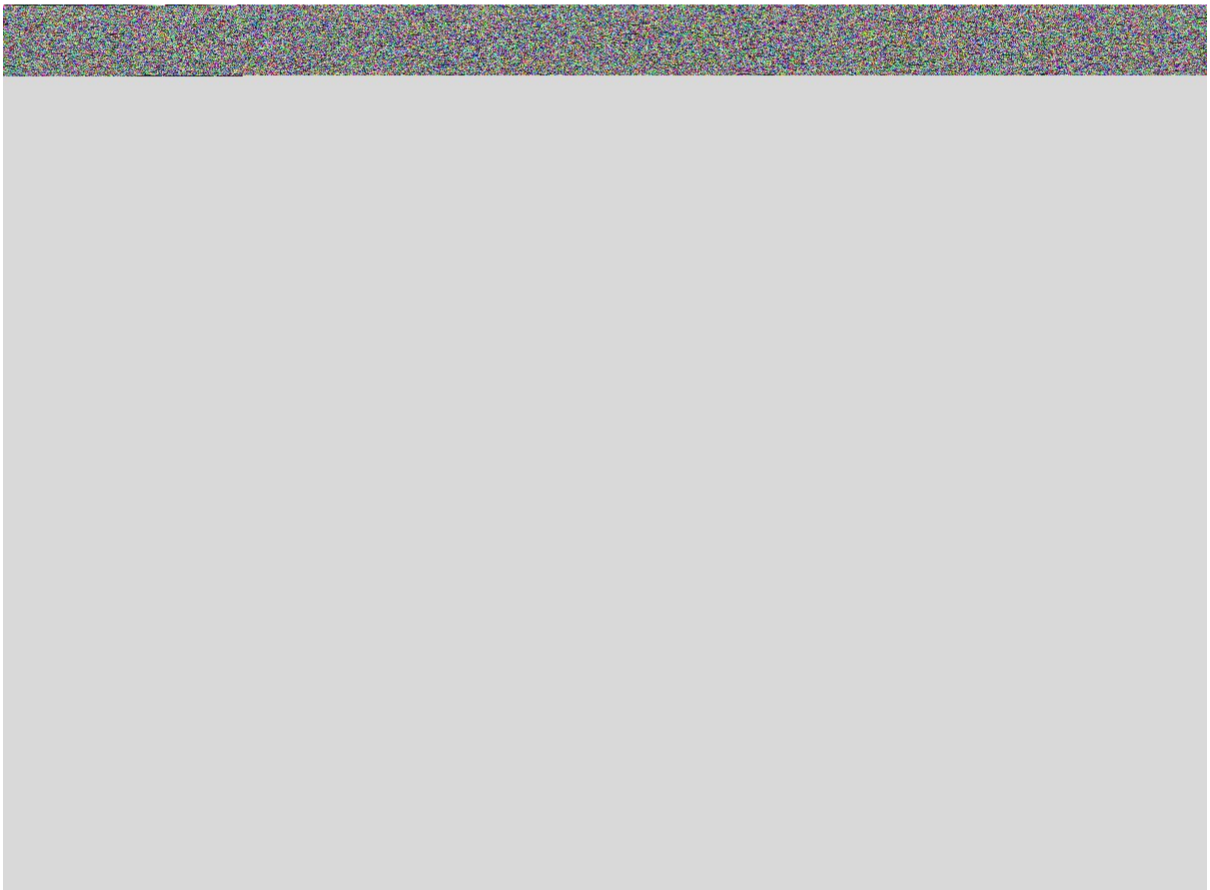

S4: Examples of loadings and scores derived from the data matrix of goat IVD samples (set one) in the spectral region of 950-1300 cm^{-1}; calculated using SVD, NIPALS as well as NIPLAS-MALS and SVD-MALS methods. All calculations are based on five components.
